# Carcinome épidermoïde du périnée masculin: à propos d´un cas

**DOI:** 10.11604/pamj.2023.44.50.38398

**Published:** 2023-01-25

**Authors:** Mamadou Lamine Diakité, Cheickna Badiaga, Aissata Samassekou, Coulibaly Seydou, Mayoro Dembélé, Honoré Jean Gabriel Berthe

**Affiliations:** 1 Service d´Urologie, Centre Hospitalier Universitaire Point-G, Bamako, Mali

**Keywords:** Carcinome épidermoïde, périnée masculin, chimiothérapie, radiothérapie, cas clinique, Squamous cell carcinoma, male perineum, chemotherapy, radiotherapy, case report

## Abstract

Le carcinome épidermoïde du périnée masculin est exceptionnel. Nous rapportons le cas d’un patient âgé 42 ans sans antécédent médical, qui avait consulté pour une gêne pelvienne depuis 4 mois. Le patient était suivi dans un centre de santé de Bamako pour abcès périnéal. L’examen anatomopathologique a confirmé le diagnostic. Le traitement est fonction du stade de la lésion et de sa localisation. Le pronostic demeure sombre. Cependant notre espoir pour ce patient réside dans des protocoles thérapeutiques combinant chimiothérapie et radiothérapie en se basant sur les résultats obtenus dans les cancers épidermoïdes de l´œsophage et de l´anus. Le but de ce travail est de rapporter un premier cas dans le service.

## Abstract (en)

Squamous cell carcinoma of the male perineum is exceptional. We report the case of a 42-year-old patient with no previous medical history, presenting with pelvic discomfort lasting for 4 months. The patient was treated for perineal abscess in a health centre in Bamako. The anatomo-pathological examination confirmed the diagnosis. Treatment depends on the stage of the lesion and its location but it is associated with a poor prognosis. Given the results achieved in patients with epidermoid cancers of the esophagus and anus, treatment was based on therapeutic protocols combining chemotherapy and radiotherapy. The purpose of this work was to report the first case in our hospital unit.

## Introduction

Le carcinome épidermoïde du périnée masculin est exceptionnel. Généralement, on distingue deux formes de carcinomes: baso-cellulaires ou épidermoïdes. Les carcinomes baso-cellulaires sont les plus fréquents (70% des cancers cutanés) et les moins graves tandis que les carcinomes épidermoïdes sont plus rares (20%) mais plus agressifs. Ils se développent à partir des couches supérieures de l’épiderme et ont la capacité d’envahir les ganglions lymphatiques et de métastaser. Il est donc essentiel de détecter ces carcinomes le plus tôt possible [1].

## Patient et observation

**Information du patient:** un patient de 42 ans sans emploi, sans antécédent particulier notable chez qui nous avons découvert une tumeur maligne rare de localisation périnéale entre le scrotum et l´anus dans le service lors de son hospitalisation. Cette découverte était fortuite au cours d´une hospitalisation pour tuméfaction et abcès du scrotum avec suspicion de rétrécissement de l´urètre. L´état général du patient était passable, il était moyennement coloré avec une légère diminution du poids corporel. Cette tumeur avait les caractéristiques cliniques suivantes: une ulcération (plaie) du périnée dure, le toucher rectal et l´examen ano-rectoscopique normaux; l´urètre normal à l´UCR; une importante infiltration des tissus sous cutanés à l´échographie. Elle prête à confusion avec la maladie de Verneuil, hydradénite suppurative qui est une maladie inflammatoire chronique, suppurative, fistulisante et d’évolution cicatricielle des régions cutanées comportant des glandes apocrines. Sa dégénérescence en carcinome épidermoïde a été répertoriée plusieurs fois dans la littérature [2].

**Résultats cliniques:** les fragments examinés sont entièrement tumoraux. La tumeur est faite de lobules, des travées et de papilles constitués de cellules polygonales avec anisocaryose, hyperchromatisme et de volumineux nucléoles. On note des mitoses anormales et des foyers de dyskératose. Le stroma est peu abondant fibro inflammatoire et nécrotique. En surface, il s´agit d´un épithélium malpighien kératinisé régulier. Ce qui conclut à un carcinome épidermoïde moyennement différencié mature et invasif du périnée. Nous rapportons un cas rare de carcinome épidermoïde du périnée masculin de localisation périnéale respectant l´urètre et l´anus.

**Figure 1:**
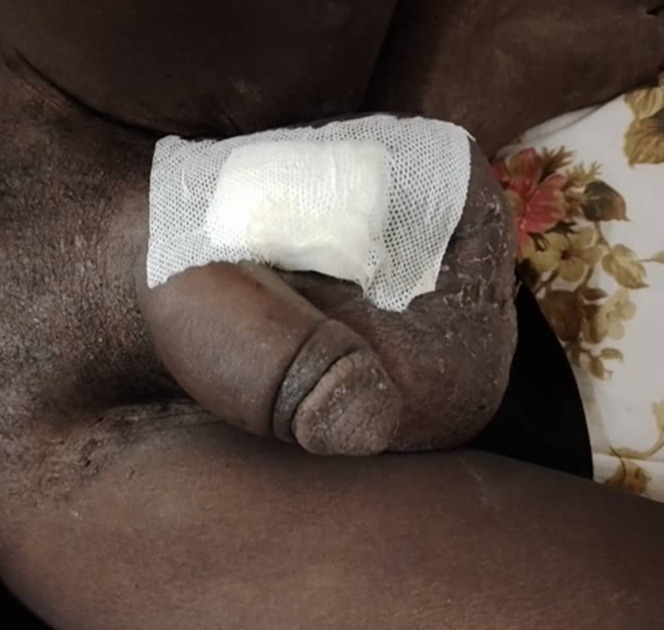
aspect avec pansement scrotal

**Figure 2:**
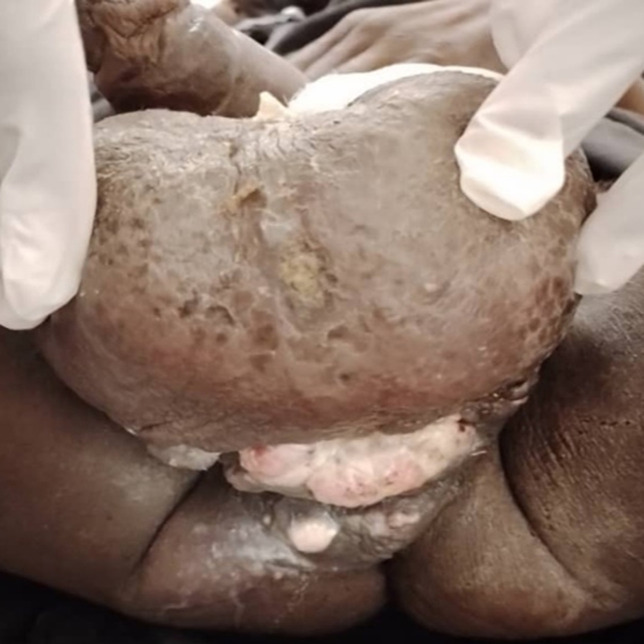
plaie du périnée après l’abcès

**Constatations cliniques et chronologie:** un patient âgé de 42 ans, sans antécédent médical, présentait depuis 4 mois une tuméfaction dure et indolore du périnée avec augmentation progressive du volume des bourses sans signes digestifs ni urinaires associés. Le tout évoluait dans un contexte d´apyrexie et d´altération progressive de l´état général. Après collection partielle, il a été pris en charge dans un centre de santé de référence pour abcès périnéal en février 2022. Devant le retard de la cicatrisation, la persistance de la tuméfaction et l´altération de l´état général; il a été référé dans le service au CHU du point-G pour meilleure prise en charge le 02 juillet 2022 (Figure 1, Figure 2).

**Evaluation diagnostique:** la biologie: un taux d´hémoglobine à 10,8 gr.; Gr./Rh. A+; Créatinémie: 100 micromol/l; Glycémie: 7 mmol/l; CRP: 92 mg/l; Iono: Normal; SRV: Négatif; ECBU: Normal. Le toucher rectal, l´échographie, l´uretro-cystographie rétrograde (UCR) et l´ano-rectoscopie n´ont pu objectiver une pathologie. A noter une absence d´adénopathie inguinale. Le patient avait une miction normale. Une biopsie exérèse de la masse a été réalisée. Histologiquement, il s´agissait d´une prolifération tumorale faite de lobules, des travées et de papilles constituées de cellules polygonales avec anisocaryose, hyperchromatisme et de volumineux nucléoles avec des mitoses anormales et des foyers de dyskératose. Le stroma est peu abondant fibro inflammatoire et nécrotique. En surface, il s´agit d´un épithélium malpighien kératinisé régulier. Conclus à un carcinome épidermoïde moyennement différencié mature et invasif du périnée. D´autres examens n´ont pu être réalisés par faute de moyen (Tableau 1). Le patient a été classé à un stade clinique T2Nx M0 [3]. En se référant sur la classification histologique de l´OMS 2004, nous pouvons classer notre carcinome épidermoïde moyennement différencié mature et invasif du périnée au Grade histologique G2 [4].

**Tableau 1:** classification TNM des carcinomes épidermoïde cutané (CEC) invasifs basée sur l’Union Internationale Contre le Cancer (UICC) (2009/2010) issue des recommandations européennes de 2015 [3]

Stade T	Description
**T- Tumeur primitive**	
T1	Tumeur < 2 cm dans sa plus grande dimension
T2	Tumeur > 2 cm dans la plus grande dimension
T3	Profondeur de la tumeur (muscles squelettiques, cartilage, os)
T4	Invasion de la base du crâne ou de la colonne vertébrale
**N- Atteinte régionale ganglionnaire**	
Nx	Atteinte ganglionnaire non évaluable
N0	Pas d´atteinte des ganglions ganglionnaires régionales
N1	Métastase ganglionnaire unique avec un diamètre maximum de < 3 cm
N2	Métastase ganglionnaire unique avec un diamètre maximum de > 3cm jusqu´à 6 cm, multiple atteinte ganglionnaire, dont le diamètre maximum ≤ 6cm
N3	Métastase ganglionnaire > 6 cm
**M- Métastases à distance**	
M0	Aucune métastase à distance
M1	Métastase à distance

**Intervention thérapeutique:** le patient est sous chimiothérapie depuis le 11 août 2022 avec l´assistance du service social.

**Suivi et résultats:** il est suivi en oncologie; à deux (2) mois de traitement, l´évolution clinique est marquée par une persistance de la tuméfaction (infiltration) scrotale et de la plaie périnéale. Le patient est actuellement à domicile, il n´est pas régulièrement prise en charge par faute de moyen financier. Nous gardons un contact téléphonique avec la famille. Aucun résultat positif pour le moment.

**Perspective du patient:** il est partant pour sa prise en charge. Malheureusement, ses pouvoirs sont limités.

**Consentement éclairé:** l´aspect éthique était respecté. L´accord préalable du patient était donné à toutes les étapes.

## Discussion

Le carcinome épidermoïde du périnée est une tumeur maligne rare, localisée au périnée, d´étiologie inconnue, appartenant, selon la classification 2021 de l´Organisation Mondiale de la Santé au code C49.5 (Tissu conjonctif et autres tissus mous du pelvis et l’aine fesse périnée) [1]. Le lipome géant pelvien est rare de localisation pré-sacré avec parfois des prolongements vers la région inguinale ou dans le périnée [5]. La tumeur de Buschke-Lowenstein ou condylome acuminé géant localisé au niveau du scrotum est aussi une maladie rare mais d´origine virale (Human Papilloma Virus) [6]. La littérature sur notre sujet est très pauvre et les seules études contrôlées portent sur des carcinomes épidermoïdes (CE) de la vulve et du canal anal qui ne peuvent être assimilés totalement aux CE cutané (CEC) [7,8]. La prise en charge de notre patient est difficile à cause d´une insuffisance de moyens. Le bilan d’extension consistait à déterminer, au moyen d’examens diagnostiques cliniques et complémentaires, le stade de la maladie. Il a pour objectif de détecter la présence de métastases visibles. Au terme de nos investigations, nous avons pu classé notre tumeur supérieure à 2 cm au stade clinique T2MxNx [3].

Avec la classification histologique de l´OMS 2004, nous pouvons classer notre carcinome épidermoïde moyennement différencié mature et invasif du périnée au Grade histologique G2 [4]. Avec l’identification du type et du grade de la tumeur cancéreuse, le reste du bilan d’extension permet de définir le pronostic de la maladie et le plan de traitement. Une surveillance est ainsi indispensable. Elle devra être assurée par le chirurgien ou l´oncologue, sachant qu´elle est difficile sur la durée avec des patients ayant tendance à être perdus de vue, car les moyens sont insuffisants pour faire face au coût de la prise en charge. Dans la littérature, pour un patient jeune; le traitement combiné de radio-chimiothérapie peut amener, dans une proportion non négligeable de cas de carcinomes épidermoïdes, une rémission complète. Cette radiothérapie associée à une chimiothérapie sensibilisante doit donc être proposée comme alternative à une chirurgie mutilante [7,9].

Ailleurs, la radiothérapie a peu de bénéfice thérapeutique dans le traitement d’un carcinome épidermoïde du scrotum [8]. Notre patient est présentement suivi en oncologie pour des séances de chimiothérapie. Le pronostic étant basé sur l´évolution habituelle de la maladie, il ne constitue pas un verdict dans ce cas. Chaque personne est différente et c´est une première découverte dans le service. Le carcinome épidermoïde du périnée et de l´urètre masculin sont différents du cystadénome papillaire de l´épididyme et du lipome paratesticulaire qui sont aussi des pathologies rares mais de bon pronostic [10].

## Conclusion

Notre espoir pour ce patient porteur d´un carcinome épidermoïde du périnée réside dans des protocoles thérapeutiques combinant chimiothérapie et radiothérapie en se basant sur les résultats obtenus dans les cancers épidermoïdes de l´œsophage, de la vulve et de l´anus. La radiothérapie fait partie intégrante de l´arsenal thérapeutique des carcinomes épidermoïdes. Elle sera particulièrement utile dans notre cas presque inopérable. Cette tumeur du périnée masculin reste d´un mauvais pronostic malgré la diversité des traitements, en effet sa rareté rend difficile l´évaluation des moyens thérapeutiques. Cependant des efforts doivent être fourni pour rendre accessible le traitement à moindre coût afin d´évaluer les résultats.
